# Increased prevalence of attention deficit hyperactivity disorder in children with Familial Mediterranean Fever

**DOI:** 10.1186/1546-0096-13-S1-P131

**Published:** 2015-09-28

**Authors:** E Lavi, I Berger, E Eisenstein, Y Berkun

**Affiliations:** 1 Hadassah-Hebrew University Medical Center, Mount Scopus, Israel, pediatrics, Jerusalem, Israel

## Introduction

Attention deficit hyperactivity disorder (ADHD) is a developmental neuropsychiatric disorder characterized by inappropriate levels of inattention, impulsivity and hyperactivity. The cause of ADHD is unknown, but may involve both genetic and environmental factors. It has been suggested that exposure to inflammation in infancy may increase the risk for ADHD in later life. Familial Mediterranean Fever (FMF) is the most common inherited autoinflammatory disorder. In many FMF patients the inflammation persists in attack-free periods. The prevalence of ADHD among FMF patients has not been studied previously.

## Objectives

To explore the prevalence of ADHD among FMF patients and to examine the relationship between FMF characteristics and ADHD.

## Patients and methods

The cohort consisted of 103 consecutive children with FMF, followed in a single referral center. Clinical manifestations, demographic and genetic data were abstracted from the patients' medical records, supplemented by information obtained by interviews conducted during routine follow up visits. The presence of ADHD was assessed using the Diagnostic and Statistical Manual of Mental Disorders questionnaire (4th ed.; DSM-IV).

## Results

ADHD was diagnosed in 33 (32.4%) FMF patients, a rate significantly higher that known in our local unselected population (about 8%). The distribution of ADHD subtypes in our patients was similar to the general population: 10 children had predominantly inattentive type (9.8%), 6 hyperactive-impulsive (5.9%) and 17 combined type (16.7%). FMF patients diagnosed with ADHD had a higher rate of arthritis and family history of FMF than patients without ADHD.

## Conclusion

The high prevalence of ADHD in children with FMF may support the neuroimmune hypothesis, in which inflammatory conditions increase the risk for ADHD. Furthermore, our findings suggest that physicians should be alert to the possible presence of ADHD among FMF patients.

